# Evolutionary consequences of epigenetic inheritance

**DOI:** 10.1038/s41437-018-0113-y

**Published:** 2018-07-05

**Authors:** Martin I. Lind, Foteini Spagopoulou

**Affiliations:** Department of Ecology and Genetics, Animal Ecology, Uppsala University, Uppsala, 752 36 Sweden

**Keywords:** Epigenetics, Evolutionary genetics, Gene regulation

## What is epigenetics and epigenetic inheritance?

In recent years, the belief that the genetic code is the sole basis for biological inheritance has been challenged by the discovery of trans-generational epigenetic inheritance. Environmentally induced phenotypes can in this way persist for several generations, due to the transmission of molecular factors that determine how DNA is read and expressed (Jablonka and Raz 2009; Bonduriansky and Day 2009). Epigenetic regulation of gene expression is a common process that acts during the differentiation of somatic cells, as well as in response to environmental cues and stresses, and the passing on of these modulations to the offspring constitutes epigenetic inheritance. While the term ‘epigenetics’ (επ? [epi] + genetics, which means ‘on top of/around of’ genetics) was first coined 76 years ago (Waddington 1942), the field has only recently gained an upsurge due to the emergence of modern sequencing methods (Verhoeven et al. 2016; Allis and Jenuwein 2016). These technological advances have highlighted the role and importance of a number of proximate mechanisms of epigenetic inheritance, including DNA methylation, histone modification and small RNA transmission.

There is an overall lack in consistency with regards to the definition of epigenetic inheritance used, but here we adopt the broad definition of Burggren (2016) and consider trans-generational epigenetic inheritance as the non-genetic inheritance of a modified phenotype across generations, without focus on a specific mechanism. This definition enables us to concentrate on the evolutionary consequences of epigenetic inheritance. The focus of this special issue is broadly on the evolutionary forces selecting for epigenetic inheritance, its costs and importance for adaptation. In particular, we specifically highlight the effects of paternal trans-generational epigenetic inheritance, which until now have received comparatively little attention.

## Epigenetics and evolution

Epigenetic effects are not only common, but can also underlie and influence many aspects of evolution. This is true for both epigenetic effects that are only expressed within a single generation, as well as for trans-generational epigenetic inheritance. In this special issue, Banta and Richards (2018) show that epigenetics can have profound influence over a number of evolutionary aspects. By using the quantitative genetic formula for the partitioning of phenotypic variance, they show that epigenetic mechanisms can underlie or influence all of its parameters. Moreover, if such epigenetic modulations are also heritable, they can be erroneously misinterpreted as genetic variance. The widespread presence of epigenetic marks in natural populations, thus, poses a challenge for researchers aiming to infer quantitative genetic parameters or estimate population divergence in quantitative traits. While it is well known that phenotypic plasticity can cause similarities between individuals that are not based upon genetic resemblance (Pujol et al. 2008), the same can also hold true for epigenetic inheritance of traits. The authors review the development of breeding designs and analyses that aim to partition epigenetic variance from total genetic variance, but also highlight that epigenetic modulations can still affect the remaining quantitative genetic parameters.

## Adaptive epigenetic inheritance

While epigenetic inheritance is well documented (Verhoeven et al. 2016), the adaptive significance, if any, of such a complementary inheritance system remains enigmatic. Since it constitutes the inheritance of an environmentally induced phenotype, its adaptive value should depend upon whether environments are predictable across generations. In a slowly fluctuating environment, where the cycle length is longer than the generation time, the parental environment may function as a reliable cue for the offspring developmental conditions, and epigenetic inheritance could therefore evolve as an anticipatory effect (Burgess and Marshall 2014). While the underlying theory is well-developed (Jablonka and Raz 2009; Uller et al. 2015), performing direct tests of this central prediction can be challenging, since it would require comparing the degree of epigenetic inheritance in populations evolving in environments predicted to select *for* or *against* epigenetic inheritance. Nevertheless, there are some phenomena that fit the predictions of these theoretical models.

In this special issue, Roth et al. (2018) review recent advances in trans-generational immune priming, one of the most well-studied examples of trans-generational inheritance. The transfer of parental immunological experience to enhance the offspring immune defence is present in both vertebrates and invertebrates, and can be inherited for multiple generations. Furthermore, recent evidence suggests that epigenetic mechanisms such as DNA methylation, histone modifications and small RNAs may underlie many of these responses. Finally, Roth et al. (2018) discuss the selection pressures leading to the evolution of trans-generational immune priming in some systems, but not in others.

## Epigenetic inheritance and evolutionary potential

Epigenetic inheritance can be important for adaptation, especially in cases where the available genetic variation is limited. Firstly, epigenetic inheritance, like phenotypic plasticity, can enable survival in new environments before genetic adaptation evolves (Burggren 2016). Secondly, the rate of spontaneous gains and losses of individually methylated sites (i.e. the epimutation rate) is estimated to be substantially higher than the genetic mutation rate (Graaf et al. 2015), creating new heritable variation that can ultimately enable adaptation. Finally, for small populations with limited genetic variation, or asexual organisms, epigenetic variation can be a major source of heritable variation that can enable adaptation to new environments.

The realisation that natural populations harbour substantial amounts of epigenetic variation (e.g. Herrera et al. 2016; Thorson et al. 2017) raises the question of what degree this epigenetic variation can contribute to phenotypic variation, the actual target of selection. In this special issue, Zhang et al. (2018) explore the evolutionary potential of such epigenetic variation, by using two populations of epigenetic recombinant inbred lines (epiRILs) of the plant *Arabidopsis thaliana*, created by crossing hypomethylation mutants to their wild type. The heritable variation in phenology, growth and fitness of these epiRILs was then compared to populations of genetic recombinant inbred lines (RILs), as well as to natural collections. They found that the epiRILs could have both within and among-line variance of similar magnitude to RILs and natural populations, highlighting the importance of epigenetic variation for phenotypic variance that could ultimately aid adaptation.

However, if no genetic variation is available, adaptation to a new selective pressure can take place either when there is selection on an epigenetically induced phenotype, or when the phenotype is recreated in every generation by phenotypic plasticity. In this issue, Sentis et al. (2018) used an experimental evolution approach to investigate the non-genetic evolution of a response to predators, in a single clone of the pea aphid *Acyrthosiphon pisum*. Exposure to ladybird predators caused a plastic reduction in the asexual production of the winged morph, but over evolutionary time and constant exposure to predators, this plasticity declined. Furthermore, there was possible evolution of a reversible epigenetic mechanism, highlighting the importance of both plasticity and epigenetic inheritance for adaptation, when limited genetic variation is present.

## Paternal epigenetic effects

Traditionally, trans-generational epigenetic inheritance was only recognised and studied via the maternal line. Paternal epigenetic inheritance had for long been considered as rare, existing only in species where males provide parental care (Kokko and Jennions 2008). This asymmetry can be largely explained by the lack of known transfer mechanisms of epigenetic molecular factors between father–offspring and the difficulty of differentiating between paternal and maternal epigenetic effects, given that paternal influence is mediated by maternal responses (Crean and Bonduriansky 2014). However, an increasing body of research, such as evidence for sperm- and ejaculate-mediated mechanisms of transfer, have now highlighted that paternal trans-generational epigenetic effects can be important and widespread (Jablonka and Raz 2009; Crean and Bonduriansky 2014). In this special issue, Immler (2018) reviews the non-genetic factors and mechanisms known to be transferred from sperm to zygote, which includes DNA methylation, histone modifications and transfer of small RNAs and proteins. Not only do epigenetic mechanisms convey information about the environment, but they may also be important players in the sexual conflict between mothers and fathers over gene expression in the offspring.

However, producing and maintaining an effective epigenetic machinery can have substantial metabolic costs, and thus only some males in a population could invest in such an efficient epigenome. In this issue, Macartney et al. (2018), going beyond the classic nutrient-centric view of parental investment, review findings which suggest that paternal trans-generational epigenetic effects should be seen as costly traits and, therefore, evolve a strong condition-dependent expression. The authors discuss under what conditions selection should act on paternal trans-generational epigenetic effects, suggest how to investigate these differential costs and condition dependence, and, finally, argue that paternal epigenetic effects should be incorporated as parental investment-traits into life-history theory.

## Trans-generational costs

In addition to the potential for adaptive transfer of environmental information across generations, there is an increased awareness that the parental environment may result in non-genetically transferred costs that are expressed in the offspring, sometimes lasting for several generations. In this special issue, Zajitschek et al. (2018) investigate the trans-generational costs of multiple mating and harassment of females over three generations, in the seed beetle *Callosobruchus maculatus*. While mothers showed neither costs nor benefits of multiple mating, the offspring of these harassed mothers had lower fecundity, an effect that was interestingly reversed in the granddaughters. Furthermore, lifespan was negatively correlated to reproduction, as predicted by life-history theory. This study highlights the importance of considering trans-generational costs when investigating life-history trade-offs, and their dynamics across generations.

## Future challenges

The study of epigenetic inheritance has moved from documenting its existence to exploring its evolutionary consequences. Of the many exciting areas of research that have now opened up, there are three key questions that we would especially like to highlight.

### Is epigenetic inheritance generally adaptive?

Models suggest that epigenetic inheritance of the parental phenotype can be adaptive in slowly fluctuating and correlated environments, since the parent and offspring will most often share the same environmental conditions (Jablonka and Raz 2009; Uller et al. 2015). However, direct tests of this prediction are, so far, lacking. On the other hand, if parents and offspring live in negatively correlated environments, the parental phenotype should not be inherited, but the parents can still anticipate the offspring environment. As such, the evolution of a non-genetic parental effect could be adaptive, a prediction that has recently received experimental support (Dey et al. 2016). This fits the broad concept of parental effects (Uller et al. 2015) and suggests that anticipatory effects (such as epigenetic inheritance) can evolve if environments are predictable across generations. Furthermore, adaptive environmentally induced epigenetic inheritance is also expected to be more common in organisms with a short life cycle than in, for example, long lived mammals (Houri-Zeevi and Rechavi 2017). To investigate these questions and gain a deeper understanding in the evolutionary dynamics of epigenetic inheritance, it is important to focus on a diverse set of study systems with known degrees of ecological variability.

### Does epigenetic inheritance influence genetic adaptation?

Epigenetic inheritance can aid adaptation in two ways. Firstly, adaptive environmentally induced epigenetic inheritance can move a population closer to a fitness peak, than if they would rely only on genetic change (Kronholm and Collins 2016). If the new environment is stable, genetic change may ultimately follow, according to the same principles as the genetic fixation of initially induced phenotypes (i.e. genetic assimilation). Secondly, because of the high epi-mutation rate (Graaf et al. 2015), selection can act upon randomly induced epigenetic variants that if they are inherited, can aid adaptation in a similar scenario as described above. It is, however, challenging to separate genetic and epigenetic variation, but work on clonal organisms that invade new environments can be a suitable starting point.

### How important is paternal epigenetic inheritance?

While we have become increasingly aware that fathers can have a large influence on trans-generational inheritance, we are far from understanding whether such paternal effects are equally as important as maternal effects. Furthermore, we should also explore and identify the importance of the ejaculate as a whole in the paternal trans-generational inheritance. Apart from sperm, males also transfer factors to females via the seminal fluid, and such factors can have long-term consequences in both females and offspring (Crean et al. 2014; Bromfield et al. 2014). Paternal trans-generational effects are mediated by female responses, which also open up a potential sexual conflict over gene expression in the offspring (Crean and Bonduriansky 2014). Experiments that aim to disentangle these effects will allow us to investigate the relative importance and mechanisms of paternal trans-generational epigenetic inheritance.

### Concluding remarks

As this set of papers illustrate, epigenetics and epigenetic inheritance can have profound influence on evolution. Trans-generational epigenetic inheritance not only needs to be taken into account when estimating quantitative genetic parameters, but it can also respond to selection and influence adaptation to new environments. Thus, the emerging field of epigenetic inheritance has many similarities with the now well-developed field of phenotypic plasticity, which has moved from being considered a nuisance, into becoming a major research field with considerable importance for adaptation. Plasticity and epigenetic inheritance can share underlying mechanisms that regulate gene expression. In both cases the degree of environmental heterogeneity and its stability over generations emerges as a determining factor influencing their evolution. The contributions to this special issue give an important snapshot of the state of the adaptive epigenetic inheritance field, highlight its evolutionary consequences and point out important directions forward.

The idea for this special issue was based in part on the symposium entitled ‘Evolutionary implications of transposable elements, epigenetics, and non-genetic inheritance’, held at the European Society for Evolutionary Biology conference in Groningen, the Netherlands in August, 2017.
